# Clinical Outcome of Pulsed-Radiofrequency Combined With Transforaminal Epidural Steroid Injection for Lumbosacral Radicular Pain Caused by Distinct Etiology

**DOI:** 10.3389/fnins.2021.683298

**Published:** 2021-07-28

**Authors:** Liuqing Yang, Yuzhao Huang, Jiahui Ma, Zhenxing Li, Rui Han, Gangwen Guo, Yuncheng Ni, Rong Hu, Xuebin Yan, Haocheng Zhou, Dong Huang

**Affiliations:** ^1^Department of Pain, The Third Xiangya Hospital and Institute of Pain Medicine, Central South University, Changsha, China; ^2^Department of Orthopedics, The Third Xiangya Hospital, Central South University, Changsha, China; ^3^Hunan Key Laboratory of Brain Homeostasis, Central South University, Changsha, China

**Keywords:** neuromodulation, pulsed radiofrequency, transforaminal epidural steroid injection, disc herniation, spinal stenosis, failed back surgery syndrome, radicular pain, chronic pain

## Abstract

**Background:**

Lumbosacral radicular pain (LSRP) can be caused by disc herniation, spinal stenosis, and failed back surgery syndrome. The clinical effect of pulsed-radiofrequency (PRF) combined with transforaminal epidural steroid injection (TESI) for radiating pain in different population remains unclear.

**Methods:**

We retrospectively reviewed the medical recordings of patients with LSRP caused by different etiologies, who underwent PRF and TESI treatment. The primary clinical outcome was assessed by a 10-point Visual Analog Scale (VAS) pre- and post-treatment.

**Results:**

A total of 34 LSRP patients were identified and classified into 3 subgroups (disc herniation, spinal stenosis, and failed back surgery syndrome). The overall immediate pain reduction was 4.4 ± 1.1 after procedure. After a median follow-up of 9.5 months, the VAS decreased from 6.5 ± 1.0 to 2.4 ± 1.9 at the last follow-up.

**Conclusion:**

PRF combined with TESI is an effective approach to treat persistent LSRP in distinct population.

## Introduction

Lumbosacral radicular pain (LSRP) is defined as a radiating pain affected one or more lumbar or sacral dermatomes (Van Boxem et al., 2010). LSRP is commonly accompanied with disc herniation (DH), spinal stenosis (SS), and failed back surgery syndrome (FBSS) (Abejón et al., 2007; Park and Lee, 2019; Clingan et al., 2020). Conservative therapy remains the first option for the initial management of LSRP, including oral NSAIDs and/or anticonvulsants, exercise, and physiotherapy. In addition, epidural corticosteroid injections may provide supplementary relief from pain at short-term follow-up (Oliveira et al., 2020). The principal goal of surgical intervention is to remove the compression of nerve root. However, no significant improvement of pain or physical function was achieved through the discectomy compared with conservative care up to 2 years follow-up (Weinstein et al., 2006). Thus, alternative option of LSRP management is further needed.

A complex interplay between mechanical, inflammatory, immune and neurophysiologic mechanism of dorsal root ganglion (DRG) attributes to the chronicity of radicular pain (Dower et al., 2019). Thus, it is essential to normalize the dysfunction of DRG to achieve sustained pain relief in patient with or without mechanical compression. Pulsed-radiofrequency (PRF) is the most widely used technique of neuromodulation in pain management. Recently, PRF adjacent to the DRG has been increasingly used to treat cervical, thoracic, lumbar, and sacral radicular pain (Koh et al., 2015; Hetta et al., 2020; Lee et al., 2020). After administration of PRF, more than three quarters of patients with lumbar radicular pain rejected to undertake spinal surgery (Trinidad et al., 2015).

Despite PRF therapy, spinal injection is another important technique to treat radicular pain when conservative therapy is ineffective. To treat chronic LSRP, different approach of epidural injections, including transforaminal epidural steroid injection (TESI) and interlaminar epidural steroid injections, can be considered. The overall percentage of the patients with significant functional improvement and pain relief receiving TESI treatment was higher than interlaminar routine (Rados et al., 2011). Given the anti-inflammatory effect, TESI may provide a supplementary analgesic effect to PRF. The aim of this study was to retrospectively evaluate the clinical efficiency of PRF combined with TESI to treat LSRP with different etiologies.

## Materials and Methods

### Study Design

**FIGURE 1:**
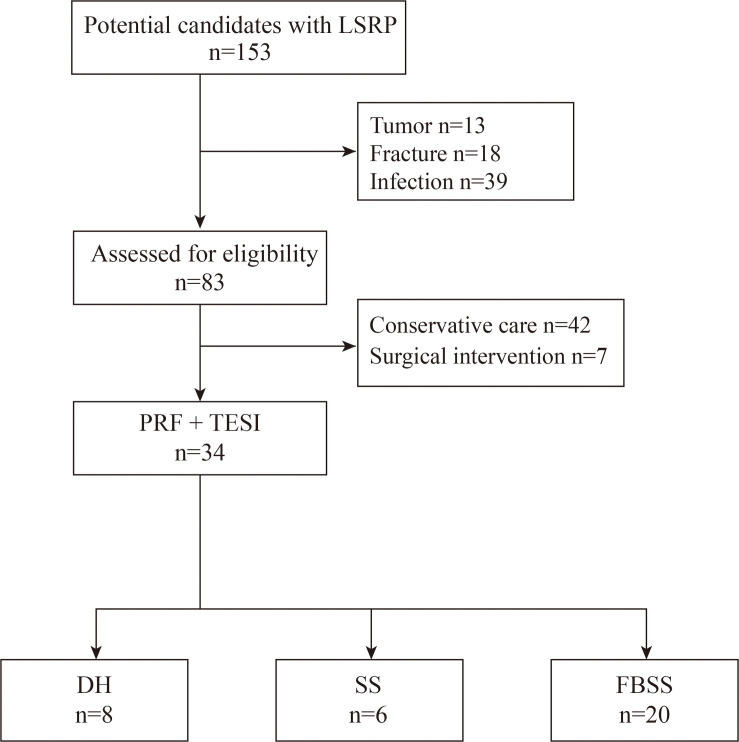
Flow chart of patient selection.

We conducted a retrospective analysis of medical recordings in the Department of Pain, the Third Xiangya Hospital between August 2019 and February 2021. All participants presented unilateral or bilateral LSRP. DH or SS was confirmed with either computed tomography or magnetic resonance imaging. We also enrolled patients present with sustained radiating pain after back surgery. One or more oral analgesic medication, or nerve block was administrated prior to surgery. This study was approved by the local ethics committee of the Third Xiangya Hospital (No. 21035) and conducted in accordance with the Declaration of Helsinki. Patients with pathologic history were excluded for further study, including tumor, fractures, and infection. The enrolled patients were subsequently divided into three subgroups according to the etiology (DH, SS, and FBSS), as shown in Figure 1. Most patients (20 out of 34) had a history of back surgery. There were 8 patients diagnosed with DH, and six for SS cohort, respectively.

### Surgical Detail

All interventional procedures were performed under the guidance of C-arm fluoroscopy (TOSHIBA). Patients were placed prone position, and kept awake during the procedure to respond the test of sensory stimulation. Local anesthesia was administrated to the skin entry with 1% lidocaine in a total volume of 5 mL.

**FIGURE 2:**
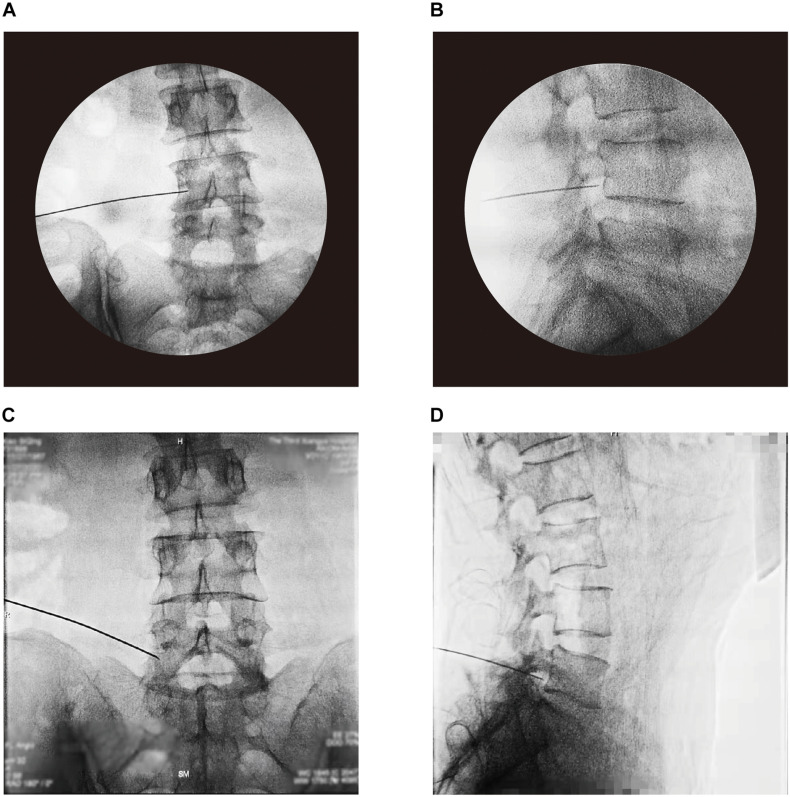
Placement of PRF cannula confirmed by the guidance of C-arm fluoroscopy. Anterior-posterior and lateral view at L4 level **(A,B)**, and L5 level **(C,D)**.

To perform PRF therapy, a 22-G curved-tip cannula was applied to place adjacent to the DRG (Figure 2). One catheter needle with active tip electrode was then inserted though the cannula, and the distal ending of electrode was connected to the radiofrequency generator (Beiqi, R-2000BA1, Beijing, China). Sensory test was conducted to induce a tingling sensation and/or dysesthesia at a voltage less than 0.5 V. PRF treatment was set at 2 Hz (20 ms pulse width) three times for 240 s. During the procedure, the temperature of electrode tip did not exceed 42°C (Tortora et al., 2021). After PRF treatment, a mixture of ropivacaine 0.2% 5 mL and betamethasone 2.5 mg was injected to the DRG site. When multiple DRGs were targeted in one procedure, the total amount of betamethasone was no more than 5 mg.

### Measurement and Follow-up

The primary outcome was the pain scores in LSRP patients. Visual analog scale (VAS) was used to evaluate the pain intensity, ranging from 0 (“pain free”) to 10 (“worst pain imaginable”). Patients who achieved reduction of pain over 50% compared with baseline were considered as “responder.” Patients were assessed pre- and post-treatment. To identify the long-term therapeutic effect, a telephone interview was conducted after discharge from hospital.

### Statistical Analyses

Statistical analysis was performed with GraphPad Prism 8 (GraphPad, San Diego, CA, United States). Variables are presented as the mean ± standard deviation. The pain score changes were assessed pre- and post-therapy with paired Student’s *t*-test. *P* < 0.05 was considered significant.

## Results

### General Demographics

We identified 34 patients (16 males and 18 females) with LSRP who underwent 57 PRF procedures combined with TESI from August 2019 until February 2021. The average age of participants was 65 ± 11.8 years. The L4-5 spinal nerve accounted for the most common lesion, affected 49 of 73 (67.1%) painful dermatomes. Patients presented moderate to severe radiating pain at admission, with a mean baseline VAS of 6.5 ± 1.0. In this study, about 76.4% patients (26 out of 34) underwent one or twice procedures during hospitalization. The general demographics of enrolled patients is given in Table 1.

**TABLE 1:** General demographics of participants.

	DH (*n* = 8)	SS (*n* = 6)	FBSS (*n* = 20)
Sex (M, F)	5, 3	2, 4	9,11
Age, median (range), years	73.5, (31–80)	72, (45–85)	62.5, (45–79)
Affected level			
Lumbar			
L2	0	1	2
L3	4	3	7
L4	7	4	17
L5	4	4	13
Sacral			
S1	1	2	3
S2	0	0	1
Pain scores before treatment, mean ± standard deviation	6.1 ± 1.2	6.7 ± 0.8	6.6 ± 1.0
Number of procedures			
1	5	4	12
2	1	1	3
3	1	1	4
4	1	0	1

### Follow-Up

All patients were evaluated pre- and post-surgery. We performed one routine follow-up 1 month after discharge from hospital for all participants. The median time of the last follow-up was 9.5 months, ranging from 1 to 18 months.

### Clinical Outcome

**FIGURE 3:**
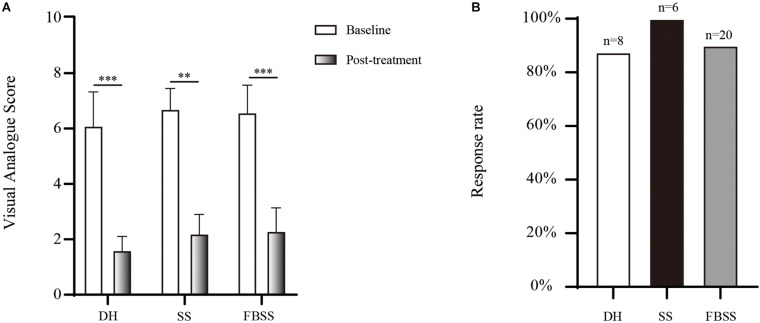
The initial analgesic effect provided by PRF and TESI. **(A)** Changes of pain score between pre- and post-treatment. **(B)** The initial response rate of each group. ^∗∗^*p* < 0.01, ^∗∗∗^*p* < 0.001.

The initial relief from pain is given in Figure 3. The immediate reduction of pain scores was 4.4 ± 1.1 across different etiologies. All SS patients achieved significant improvement of pain symptom, with pain scores reduction over 50%. The response rate before discharge was 87.5% for DH cohort, and 90% for FBSS, respectively.

**FIGURE 4:**
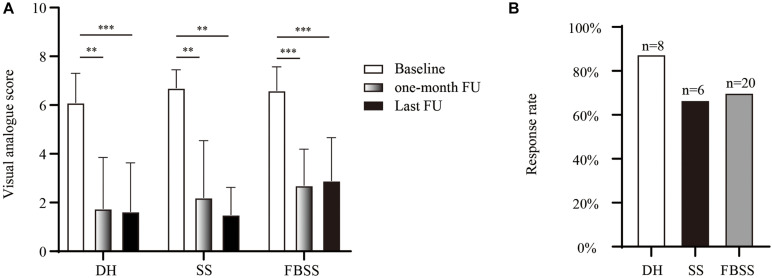
The long-term therapeutic effect. **(A)** Comparison of pain degree assessed by visual analog scores. **(B)** The long-term response rate of the last follow-up. ^∗∗^*p* < 0.01, ^∗∗∗^*p* < 0.001.

Next, we compared the effect of PRF and TESI on pain scores between different groups at 1-month after discharge and the last follow-up (Figure 4). In general, the VAS decreased from 6.5 ± 1.0 to 2.4 ± 1.9 at the last follow-up. No significant difference of pain scores was observed between 1-month and the last follow-up across all etiologies. At the last follow-up, the long-term response rate was only 66.7% for SS population, and 70% for FBSS, respectively. The combination of PRF and TESI therapy provided an enduring pain relief up to the last follow-up in DH subgroup, with 87.5% of patients presented over 50% pain relief compared with baseline.

## Discussion

LSRP is common and results from diverse etiologies, including DH, SS, and FBSS. One general feature of LSRP is sensitization of spinal nerve root or DRG, caused by mechanical, inflammatory, immune and neurophysiologic factors. Thus, novel neuromodulation therapy targeted DRG may provide an alternative option to treat LSRP.

The most common cause of LSRP is DH (Dydyk et al., 2021). However, only 24% (8/34) of patients were diagnosed with DH in this study. Majority of patients were those still presented radiating pain after spinal surgery. Similarly, the high incidence of longstanding pain with or without radicular pain ranged 20–40% in FBSS population (Thomson, 2013; Baber and Erdek, 2016). In addition, we found that over half of the participants experienced radicular pain associated with L4-5 distribution, which is consistent with previous data (DeFroda et al., 2016).

In recent years, radiofrequency therapy has evolved as a promising neuromodulatory technique for the management of various chronic pain syndromes, like radicular pain, trigeminal neuralgia, occipital pain, shoulder and knee pain (Vanneste et al., 2017; Huang et al., 2020). In current study, we applied PRF adjacent to the DRG to achieve relief from radicular pain caused by different etiologies. To our knowledge, only one study compared the clinical outcome of pulsed-radiofrequency for LSRP across different etiologies. Previous data indicated that PRF treatment was significantly more efficacious in DH and SS population than those with FBSS (Abejón et al., 2007). However, we did not observe any superior therapeutic effect in DH or SS cohort compared with FBSS in this study. One possible reason is the additional analgesic effect provided by TESI. Similarly, previous data has demonstrated that PRF combined with TESI achieved better clinical outcome of lumbar DH than PRF alone 1 month after procedure (Ding et al., 2018). Despite relief from pain, PRF can achieve significant functional improvement, demonstrated by the reduction of Oswestry Disability Index scores (Sansone et al., 2020). One significant limitation of this study was that we did not perform functional evaluation pre- and post-treatment systematically.

TESI is more specific and selected nerves can be targeted during treatment, compared with interlaminar or caudal route access. The degree of nerve root compression is the key factor to predict the long-term therapeutic effect (Ghahreman and Bogduk, 2011). Thus, one principle of our treatment is to initially identify and remove the severe compression responsible for the painful distribution. As a result, we recommended 7 patients for decompressive surgery (Figure 1) and none presented sustained radiating pain after procedure. One key factor of therapeutic effect is the ingredients present in the TESI, we administrated betamethasone during surgery based on our clinical routine. However, previous data has demonstrated that patient responded better to triamcinolone compared with betamethasone with TESI for LSRP (McCormick et al., 2015). It is essential to examine the superiority of different agents for TESI combined with PRF in the future study.

Meanwhile, patient who underwent spinal surgery may still experience pain due to previous sensitization of the nerve root (Dower et al., 2019). TESI may attenuate the pain symptom of FBSS patient by reducing the inflammatory process and desensitizing the inflamed nerve root, namely the “battered” root syndrome (Waisbrod and Gerbershagen, 1985; Ragab and Deshazo, 2008). Thus, we propose that the battered sensory nerve (nerve root and/or DRG) syndrome (BSNS) is the common pathological condition of LRSP, caused by distinct etiology. The chronicity of neuropathic pain caused by central or peripheral sensitization is the characteristic feature of BSNS.

One novel strategy of neuromodulation for LRSP is electrical DRG or spinal cord stimulation, which is most widely applied in patients with FBSS (Atallah et al., 2008; Kallewaard et al., 2019). Unlike PRF, functional electrical stimulator generates currents flow between the cathode and anode to interrupt the abnormal processing of pain signal. However, few patients can afford an expensive permanent stimulation device, and the risk of complication (e.g., lead migration, infection, and tolerance) increases with implantation duration (Eldabe et al., 2016). The mean total health care costs for the spinal cord stimulation treatment were almost 5 times more than those with conventional medical management (Manca et al., 2008). An obvious advantage of PRF or TESI is that the therapy can be repeated at low cost if necessary.

Current study has several main limitations. Firstly, the clinical data were collected retrospectively and the number of DH, and SS subgroup was smaller than FBSS. Actually, we found more patients presented intractable radicular pain with previous back surgery, compared with non-surgical population. Further study with larger cohort is needed to confirm the incidence of LRSP for individual etiology. Second, the uncontrolled nature of study design. Previous study has demonstrated the clinical efficacy was changed in different therapies (TESI vs. PRF vs. TESI combined PRF) (Ding et al., 2018). However, the superiority of each method in management of LRSP with distinct etiology remains unclear. It is essential to perform prospective, controlled study in the future to confirm the optimal indication of neuromodulation therapy.

## Conclusion

In summary, PRF combined with TESI is an effective approach to treat LSRP in patients, providing considerable sustained relief from pain with distinct etiology.

## Data Availability Statement

The data are not publicly available due to privacy and ethical reasons. The data presented in this study are available on reasonable request from the corresponding author.

## Ethics Statement

The studies involving human participants were reviewed and approved by the ethics board of The Third Xiangya Hospital, Central South University (No. 21035). The patients/participants provided their written or verbal informed consent to participate in this study.

## Author Contributions

HZ and DH: conceptualization, investigation, supervision, project administration, and funding acquisition. LY, YH, RHa, GG, YN, RHu, XY, DH, and HZ: methodology. LY, YH, and ZL: formal analysis. LY, ZL, and HZ: data curation. YH and HZ: writing—original draft preparation. HZ: writing—review and editing. All authors have read and agreed to the published version of the manuscript.

## Conflict of Interest

The authors declare that the research was conducted in the absence of any commercial or financial relationships that could be construed as a potential conflict of interest.

## Publisher’s Note

All claims expressed in this article are solely those of the authors and do not necessarily represent those of their affiliated organizations, or those of the publisher, the editors and the reviewers. Any product that may be evaluated in this article, or claim that may be made by its manufacturer, is not guaranteed or endorsed by the publisher.
